# Maternal dietary vitamin A and polyunsaturated fatty acids intake and risk of preterm birth: a nested case-control study

**DOI:** 10.7717/peerj.21068

**Published:** 2026-04-01

**Authors:** Lei Cao, Zifu Wang, Yuqing Li, Chenyan Yang, Hongchuan Fu, Qinqin Tian, Liping Yang, Baohong Mao

**Affiliations:** 1 Department of Medical Education, Gansu Provincial Maternity and Child-care Hospital (Gansu Provincial Central Hospital), Lanzhou, Gansu, China; 2 School of Public Health, Gansu University of Chinese Medicine, Lanzhou, Gansu, China; 3 Department of Medical Quality Control, Gansu Provincial Hospital of Traditional Chinese Medicine, Lanzhou, Gansu, China; 4 Department of Clinical Epidemiology, School of Public Health, Gansu University of Chinese Medicine, Lanzhou, Gansu, China

**Keywords:** Vitamin A, PUFAs, Dietary intake, PTB, A nested case control study

## Abstract

**Background:**

The impact of maternal dietary intake of vitamin A and polyunsaturated fatty acids (PUFAs) on preterm birth (PTB) risk remains uncertain. This study aims to investigate the effects of preconception and pregnancy dietary intake of these nutrients on PTB risk and to examine potential interactions between vitamin A and PUFAs.

**Methods:**

The study subjects were pregnant women who registered at the Perinatal Medicine Center of Gansu Provincial Maternity and Child-care Hospital between March 2018 and March 2019 and whose birth outcomes could be followed up. Following ethics approval and consent, one-on-one pregnancy diet surveys established a total dietary intake database for statistical analysis. PTB was used as the outcome variable, and intake levels of different substances during pregnancy were used as independent variables. Logistic regression models were employed to calculate odds ratios (OR) and their 95% confidence intervals (CI) to analyze the impact of various substance intake levels on PTB.

**Results:**

A total of 8,897 pregnant women were enrolled in this study, comprising 880 cases and 8,017 controls. Multivariate logistic regression analysis indicated that when dietary intake of vitamin A and PUFAs levels w as too low in the third trimester, the risk of PTB would be significantly increased. The specific results were (OR = 1.440, 95% CI [1.221–1.697]) and (OR = 1.319, 95% CI [1.113–1.563]), respectively. Similar conclusions were drawn in the gestational weeks subgroup. In addition, a U-shaped association was found in the third trimester of pregnancy between the intake level of dietary PUFAs and the risk of PTB, meaning that both excessive and insufficient intake increase the risk of PTB. Interaction analysis revealed that dietary vitamin A and PUFAs intake had a multiplicative interaction effect on the risk of PTB during preconception and pregnancy.

**Conclusion:**

An increased risk of PTB is associated with low intake of vitamin A and PUFAs both pre-conception and during pregnancy. Furthermore, a nonlinear relationship between intake levels and PTB risk was observed (*P* < 0.05). To reduce the risk of PTB, it is recommended that women maintain adequate dietary intake of vitamin A and PUFAs both preconception and throughout pregnancy.

## Introduction

According to the standards set by the WHO, preterm birth (PTB) is defined as delivery that occurs before 37 weeks of pregnancy (Ohuma et al., 2023). PTB is one of the leading causes of neonatal morbidity and mortality worldwide. Globally, PTB rates range from 9.3% to 12.6% of births, varying by region and country (Ahmed, Abushama & Konje, 2023; Chawanpaiboon et al., 2019). About 90% of PTB occurs in low- and middle-income countries (Azhar et al., 2025; Swarray-Deen et al., 2024). Preventing PTB holds immeasurable importance for the fetus, families, and society as a whole (Sakata et al., 2025; Yadav & Lee, 2023; Drommelschmidt et al., 2025; Lakshmanan et al., 2022; Kovács et al., 2023; Sarda et al., 2023). However, its pathogenesis is complex and influenced by multiple factors, including genetic and environmental components, as well as inflammation, infection, and maternal nutritional status (Liu & Gao, 2021). In recent years, the role of maternal nutritional status in pregnancy outcomes has garnered increasing attention. Among them, vitamin A and PUFAs may influence PTB risk by modulating immune responses, inflammation, and fetal development pathways.

Vitamin A, a fat-soluble vitamin, plays a critical role in embryonic development, immune regulation, and placental function maintenance (Carazo et al., 2021; Chapman, 2012). Epidemiological studies indicate that vitamin A deficiency may be significantly associated with adverse pregnancy outcomes, while excessive supplements may exhibit teratogenic effects (Ishaq et al., 2024; Ma et al., 2023; Abadie et al., 2023; Olson, Ameer & Goyal, 2023). This “double-edged sword” effect suggests that maintaining vitamin A homeostasis during pregnancy is crucial for pregnancy outcomes, though its association with PTB risk requires further research validation.

Among PUFAs, the ω-3 series (such as DHA and EPA) and ω-6 series (such as arachidonic acid) play critical roles in regulating inflammatory responses, maintaining cell membrane homeostasis, and signaling pathways related to parturition initiation (Kapoor et al., 2021). Maintaining a balance of PUFAs during pregnancy is of utmost importance, as insufficient intake may hinder normal fetal development (Basak & Duttaroy, 2023), while excessive intake can increase the risks of oxidative stress and lipid peroxidation (Negre-Salvayre et al., 2022; Prisacaru, 2016). Current research findings on the effects of ω-3 and ω-6 PUFAs on the risk of PTB are inconsistent (Sun et al., 2022; Serra et al., 2021; Middleton et al., 2018), and further studies are needed to confirm and clarify these conclusions.

Furthermore, the interaction between vitamin A and PUFAs in the risk of PTB remains unclear. Therefore, systematically investigating the association of these nutrients with PTB risk will allow us to analyze their independent and combined effects, and to inform the development of clinical prenatal nutritional strategies for improving pregnancy outcomes.

## Materials & Methods

### Study population

**Figure 1:**
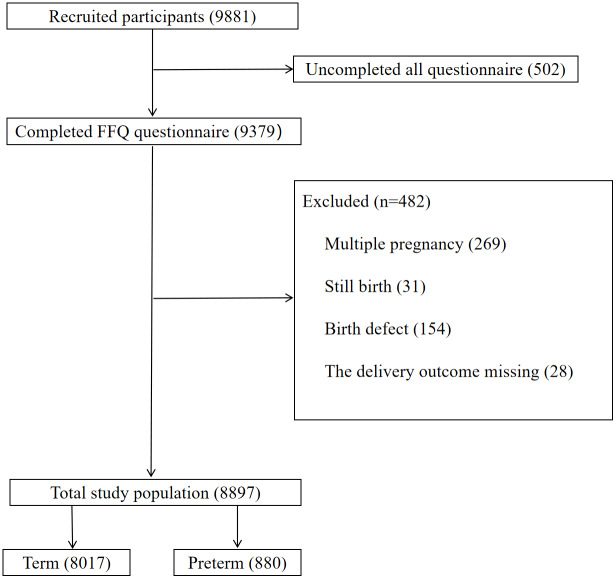
Flow chart of the study.

A birth cohort study was conducted between January 2018 and June 2019 at the largest hospital of its kind in Lanzhou City, Gansu Province. Eligible participants were pregnant women aged 18 years or older, without a history of mental illness, who received regular prenatal care and provided written informed consent. As shown in Fig. 1, the participants excluded those who did not complete the questionnaire, multiple births, still births, birth defect, and those with lost birth outcomes. The study included a total of 8,897 eligible women, comprising 880 who delivered preterm infants and 8,017 who delivered at term. The study protocol was approved by the Institutional Review Board of Gansu Provincial Maternity and Child-care Hospital [2018(029)].

### Dietary surveys, vitamin A, PUFAs daily intake

Portions of this text were previously published as part of a preprint (Wang et al., 2025). This study employed a combination of the Food Frequency Questionnaire (FFQ) and three consecutive 24-hour dietary recalls to comprehensively collect data on pregnant women, providing reliable data support for systematically analyzing factors influencing the risk of PTB. The daily dietary intake of vitamin A and PUFAs was assessed by a validated face-to-face FFQ (Huang et al., 2024; Yang et al., 2025; Ding et al., 2025). Trained researchers designed the questionnaire and collected data in strict accordance with the study protocol. All interviews were conducted by trained healthcare professionals following standardized procedures. This questionnaire collected multi-dimensional data, including: sociodemographic characteristics, medical and reproductive history, as well as detailed dietary information. This study employed three consecutive 24-hour dietary recall method for dietary assessment, covering 12 major food categories (cereals, oils/fats, vegetables, fruits, poultry, red meat products, eggs, seafood, legumes/soy products, dairy, mushrooms/seaweed, and processed snacks/beverages) and 59 specific food items.

This study employed a nested case-control design within an existing birth cohort. Since the FFQ survey is retrospective, recall bias is inevitable. To minimize recall bias, this study retrospectively collected the 24-hour dietary intake of pregnant women over three consecutive days through face-to-face interviews by professionals. During the on-site investigation, informed consent was obtained from the participants and strict quality control was implemented, including on-site review and double-entry of data by two researchers, to ensure the reliability and accuracy of the data. Additionally, medical records were reviewed to verify the diagnosis of PTB, aiming to reduce misclassification bias (Huang et al., 2024).

To obtain comprehensive dietary data for each stage, this study implemented a rigorous nutritional assessment protocol to ensure the systematic collection of relevant information throughout the entire pregnancy (Huang et al., 2024). Dietary assessments were conducted for each participant at four key stages after obtaining informed consent: (1) during preconception counseling and health examinations (within 3 months preconception); (2) during the first trimester (≤13 weeks); (3) during the second trimester (14–27 weeks); (4) during the third trimester (≥28 weeks). All three of these assessments were carried out during the scheduled prenatal check-ups at the hospital. Following completion of all dietary surveys, we quantified daily intake of vitamins and trace elements for each participant across different gestational periods using the standardized Chinese Food Composition Table (2009, 2nd edition). Nutrient intake levels were then categorized according to the Recommended Nutrient Intake (RNI) values established for Chinese residents, where RNI represents the daily intake level sufficient to meet requirements for 97.5% of the healthy population. Data on pregnancy-related complications and delivery outcomes were obtained from medical records.

### Preterm birth

Preterm birth (PTB) is defined as a live birth occurring before the completion of 37 weeks of gestation, and such cases were included in the case group for analysis. Non-PTB, defined as delivery at or beyond 37 weeks of gestation (full-term delivery), was included in the control group for analysis. Deliveries were further stratified into three categories: <32 weeks, ≤32–<34 weeks, and ≤34–<37 weeks.

### Covariates

This study implemented multi-step quality control from field investigation to data entry, including real-time review and on-site completion of questionnaires, exclusion of incomplete questionnaires, and supplementation of a small number of missing values through medical record review or telephone follow-up, thereby ensuring the integrity and accuracy of the final analytical data. The study covariates were as follows: (1) socio-demographic characteristics, including maternal age, maternal nation (Han, Hui and Minority), body mass index (BMI) during pregnancy (underweight, normal weight, overweight, and obesity), gestational weight gain, monthly income (≤2,000, 2,000∼4,000, ≥4,000 RMB *per capita*), maternal education (below middle school, above middle school and below college, above college), smoking during pregnancy (passive and active), alcohol consumption during pregnancy, and maternal employment (No/Yes). Passive smoking was defined as exposure to another person’s tobacco smoke for more than 15 min/day. (2) Maternal health-related factors including multivitamin supplementation (No/Yes), anemia during the first trimester diagnosed by the physicians using the criteria of hemoglobin concentration lower than 110 g/L, gestational diabetes (No/Yes), gestational hypertension (No/Yes), parity (primipara or multipara), abortion history, history of PTB, and neonatal sex.

### Statistical analysis

We compared selected characteristics between women with PTB and those with term birth using the chi-square test or Fisher’s exact test. The differences in measurement data between the two groups were analyzed by independent-sample t test, and the variables that did not meet the normal distribution were compared between groups by the Wilcoxon rank-sum test. Unconditional logistic regression was used to determine the odds ratios (ORs) and 95% confidence intervals (CIs) for the association between dietary vitamin A and PUFAs intake and the risk of PTB and its clinical subtypes (according to gestational weeks). Confounding factors, including weight gain during pregnancy, maternal BMI, maternal nation, maternal, gestational diabetes, total energy intake, monthly income per capita, maternal education level, smoking, maternal employment, multivitamin supplements, gestational hypertension, history of PTB, and reproductive history, were adjusted for in the unconditional logistic regression models. We used statistical software to calculate the cutoff values for vitamin A and PUFAs during preconception, first trimester, second trimester, third trimester (Li et al., 2026; Wang et al., 2026). Multivariable logistic regression analysis was performed using SAS 9.4. Nutrient intake levels at each period were categorized into high and low exposure groups based on cutoff values. OR and 95% CI for the association between each nutrient and PTB risk were calculated for each period, and the interaction between vitamin A and PUFAs was evaluated further. To explore potential nonlinear associations, multivariate-adjusted restricted cubic spline (RCS) models with three knots were applied. The number of knots was determined by comparing the criteria between Bayesian and Akaike information. The RCS models were adjusted for the potential confounding factors listed above. The multiplicative interaction parameters (*OR*=* OR11/(OR*01 ×* OR10)*) and 95% CI were also estimated by including the abovementioned variables. All statistical tests were two-sided. In data analysis, SAS 9.4 (SAS Institute, Inc., Cary, NC, USA) boasts powerful statistical capabilities and can be used for logistic regression analysis, enabling efficient exploration of the association between independent variables and categorical dependent variables. R software version 4.4.2, relying on the rich plotting packages (package ‘Hmisc’, ‘rms’, and ‘survival’), can be used to draw RCS models, visually presenting the dose–response relationship between variables.

## Results

### Basic characteristics of the study population

Among our research subjects, there were a total of 8,897 women, of whom 880 belonged to the group that gave birth to premature infants. The basic characteristics of the research subjects were shown in Table 1. Compared with women who gave birth to full-term infants, women who gave birth to premature infants had greater age distribution and ethnic differences, relatively lower educational levels, higher BMI, more smokers, higher risks of gestational diabetes and gestational hypertension, and a history of premature birth and childbirth. The two groups were similar in terms of drinking during pregnancy, anemia during pregnancy, history of abortion and the sex of the newborn.

**Table 1:** Distributions of selected characteristics of the study population.

Characteristics	Term (8,017)	Preterm (880)	*χ* ^2^	*P*-value
Maternal age			88.6426	<0.001
<23	504 (6.29)	110 (12.50)		
23 ∼<35	6,808 (84.92)	640 (72.73)		
≥35	705 (8.79)	130 (14.77)		
Maternal nation			8.473	0.015
Han	7,518 (93.78)	803 (91.25)		
Hui	289 (3.60)	46 (5.23)		
Other minority	210 (2.62)	31 (3.52)		
Maternal education level			179.736	<.001
≤Middle school	1,577 (19.67)	333 (37.84)		
Middle school-College	3,215 (41.10)	340 (38.64)		
≥College	3,225 (40.23)	207 (23.52)		
Pre-pregnancy BMI			7.693	0.021
<18.5	1,709 (21.32)	176 (20.00)		
18.5∼24	5,450 (67.98)	583 (66.25)		
≥24	858 (10.70)	121 (13.75)		
Per capita monthly income			88.84	<.001
≤2,000	1,945 (24.26)	340 (38.64)		
2,000∼4,000	4,187 (52.23)	412 (46.82)		
≥4,000	1,885 (23.51)	128 (14.55)		
Smoking (passive and active)			7.853	0.005
No	6,433 (80.24)	671 (76.25)		
Yes	1,584 (19.76)	209 (23.75)		
Drink during pregnancy			1.723	0.189
No	8,005 (99.85)	877 (99.66)		
Yes	12 (0.15)	3 (0.34)		
Anemia during pregnancy			0.869	0.351
No	7,123 (88.85)	791 (89.89)		
Yes	894 (11.15)	89 (10.11)		
Gestational diabetes			4.89	0.027
No	7,943 (99.08)	865 (98.30)		
Yes	74 (0.92)	15 (1.70)		
Gestational hypertension			267.887	<.001
No	7,726 (96.37)	738 (83.86)		
Yes	291 (3.63)	142 (16.14)		
History of preterm			138.781	<.001
No	7,910 (98.67)	818 (92.95)		
Yes	107 (1.33)	62 (7.05)		
Abortion history			0.944	0.331
No	6,919 (86.30)	749 (85.11)		
Yes	1,098 (13.70)	131 (14.89)		
Production history			60.898	<.001
No	5,983 (74.63)	549 (62.39)		
Yes	2,034 (25.37)	331 (37.61)		
Newborn’s sex			0.3922	0.531
Male	4,211 (52.53)	443 (53.64)		
Female	3,726 (47.47)	382 (46.36)		

### Associations of maternal dietary vitamin A intake with the risk of Preterm

As shown in Table 2, women with low preconception vitamin A intake exhibited a significantly higher risk of PTB compared to the high-intake group (adjusted OR = 1.194, 95% CI [1.020–1.396]). This association demonstrated a dose–response relationship across gestational periods, with progressively stronger effects observed in stratified analyses (adjusted OR = 1.343, 95% CI [1.141–1.914]; OR = 1.378, 95% CI [1.169–1.624]; and OR = 1.440, 95% CI [1.221–1.697], respectively). The risk elevation was particularly pronounced for subgroup <32 weeks (adjusted OR = 2.108, 95% CI [1.487–2.989]) and 34–37 weeks (adjusted OR = 1.317, 95% CI [1.073–1.618]) births, with the strongest effects observed during third-trimester exposure. After removing the variable “history of PTB” from the set of adjusted confounding factors, the results remained virtually unchanged, indicating robustness. Furthermore, the study found that a decrease of one quartile in vitamin A intake was associated with an increased risk of PTB, and this pattern remained consistent across all gestational age subgroups.

**Table 2:** Associations of maternal dietary vitamin A intake with the risk of preterm.

Dietary Vitamin A intake(μg/d)		Term (8,017)	Preterm (880)	OR^a^ (95% CI)	OR^b^ (95% CI)	OR^c^ (95% CI)	32<gestweek (162)			32≤gestweek<34 (170)			34≤gestweek<37 (548)		
							Cases	OR^b^ (95% CI)	OR^c^ (95% CI)	Cases	OR^b^ (95% CI)	OR^c^ (95% CI)	Cases	OR^b^ (95% CI)	OR^c^ (95% CI)
Preconception	<470.86	4,234	538	1.405 (1.219–1.621)	1.194 (1.020–1.396)	1.178 (1.008–1.377)	111	1.570 (1.099–2.243)	1.567 (1.098–2.235)	110	1.237 (0.880–1.739)	1.225 (0.872–1.722)	317	1.080 (0.892–1.307)	1.065 (0.881–1.288)
	≥470.86	3,783	342	1.000	1.000	1.000	51	1.000	1.000	60	1.000	1.000	231	1.000	1.000
	Per interquartile decrease			1.153 (1.082–1.227)	1.066 (0.994–1.144)	1.073 (1.000–1.151)		1.157 (0.990–1.353)	1.206 (1.030–1.411)		1.086 (0.933–1.264)	1.057 (0.908–1.230)		1.034 (0.948–1.127)	1.040 (0.954–1.133)
	P for trend			<.0001	0.075	0.050		0.066	0.020		0.289	0.476		0.454	0.376
First trimester	<415.05	2,200	339	1.657 (1.434–1.914)	1.343 (1.141–1.914)	1.319 (1.121–1.551)	68	1.435 (1.016–2.028)	1.411 (1.001–1.991)	71	1.414 (1.007–1.986)	1.398 (0.997–1.960)	200	1.317 (1.074–1.614)	1.299 (1.062–1.590)
	≥415.05	5,817	541	1.000	1.000	1.000	94	1.000	1.000	99	1.000	1.000	348	1.000	1.000
	Per interquartile decrease			1.237 (1.161–1.318)	1.133 (1.052–1.220)	1.126 (1.043–1.215)		1.232 (1.044–1.455)	1.207 (1.026–1.419)		1.166 (0.994–1.369)	1.156 (0.984–1.358)		1.102 (1.007–1.207)	1.099 (1.000–1.209)
	P for trend			<.0001	0.001	0.003		0.014	0.023		0.060	0.077		0.035	0.051
Second trimester	<421.26	2,156	339	1.703 (1.474–1.968)	1.378 (1.169–1.624)	1.348 (1.145–1.588)	68	1.479 (1.044–2.095)	1.472 (1.041–2.081)	65	1.216 (0.861–1.717)	1.199 (0.850–1.692)	206	1.433 (1.169–1.758)	1.410 (1.151–1.727)
	≥421.26	5,861	541	1.000	1.000	1.000	94	1.000	1.000	105	1.000	1.000	342	1.000	1.000
	Per interquartile decrease			1.232 (1.157–1.313)	1.123 (1.042–1.210)	1.129 (1.046–1.220)		1.169 (0.991–1.379)	1.177 (1.000–1.385)		1.146 (0.975–1.346)	1.120 (0.954–1.3160		1.109 (1.012–1.215)	1.130 (1.027–1.243)
	P for trend			<.0001	0.002	0.002		0.064	0.050		0.098	0.167		0.027	0.012
Third trimester	<415.49	2,083	339	1.785 (1.545–2.063)	1.440 (1.221–1.697)	1.410 (1.197–1.660)	80	2.108 (1.487–2.989)	2.077 (1.467–2.940)	67	1.367 (0.969–1.929)	1.343 (0.953–1.892)	192	1.317 (1.073–1.618)	1.297 (1.057–1.591)
	≥415.49	5,934	541	1.000	1.000	1.000	82	1.000	1.000	103	1.000	1.000	356	1.000	1.000
	Per interquartile decrease			1.258 (1.180–1.340)	1.149 (1.067–1.238)	1.193 (1.105–1.288)		1.422 (1.197–1.689)	1.604 (1.363–1.888)		1.149 (0.979–1.350)	1.124 (0.957–1.320)		1.089 (0.995–1.193)	1.112 (1.010–1.223)
	P for trend			<.0001	<.001	<.0001		<.0001	<.0001		0.089	0.154		0.064	0.030

**Notes.**

OR^a^ univariate analyses.

OR^b^ adjusted for weight gain during pregnancy, mother’s BMI, mother’s nation, mother’s age, gestational diabetes, total energy intake, monthly income per capita, maternal education level, smoking, maternal employ, multivitamin supplement, gestational hypertension, history of premature birth, reproductive history and dietary PUFAs intake.

OR^c^ was adjusted for the factors included in OR^b^, with the history of premature birth excluded.

**Figure 2:**
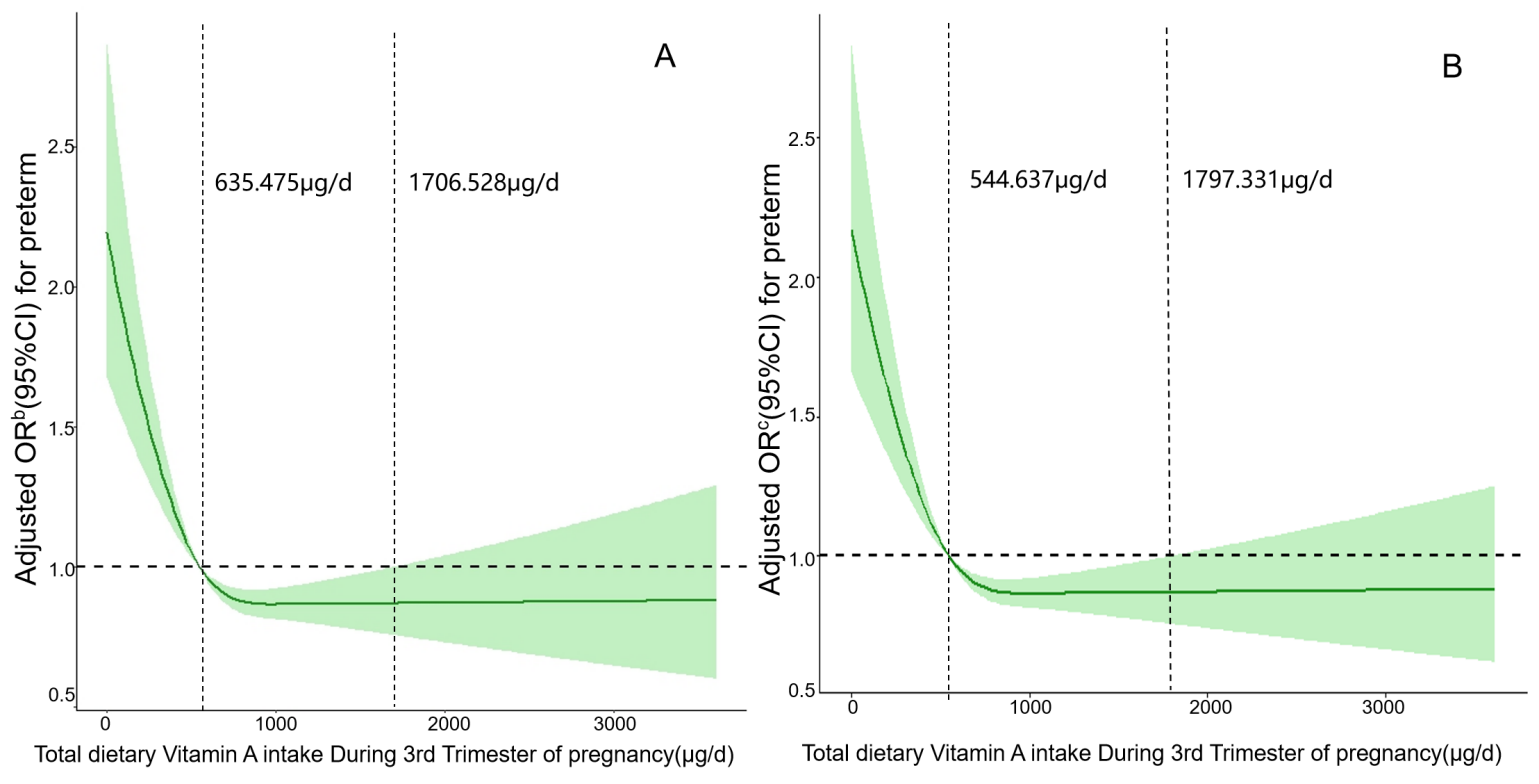
(A) and (B) respectively depict restricted cubic spline curves of the association between vitamin A intake and the risk of PTB at the third trimester before and after excluding a history of preterm birth.

Figures 2A and 2B respectively depict restricted cubic spline curves of the association between vitamin A intake and the risk of PTB at the third trimester before and after excluding a history of PTB. With the increase of daily vitamin A intake, the risk of PTB decreased rapidly and then leveled off. Vitamin A in the third trimester of pregnancy within the ranges of (635.475–1,706.528) μg/d and (544.637–1,797.331) μg/d may be a protective factor for the prevention of PTB (*P*_Nonlinear_ < 0.05). When the intake of vitamin A is respectively lower than 635.475 μg/d and 544.637 μg/d or higher than 1,706.528 μg/d and 1,797.331 μg/d, the risk of developing PTB increases.

### Associations of maternal dietary PUFAs intake with the risk of preterm

As demonstrated in Table 3, while preconception PUFAs intake showed no significant association with PTB risk, stratified analysis revealed important findings. During the third trimester, women with low PUFAs intake exhibited a 31.9% higher risk of PTB compared to those with high intake (adjusted OR = 1.319, 95% CI [1.113–1.563]). This association was particularly evident in clinically critical subgroups: for <32 weeks, the risk increased by 69.0% (adjusted OR = 1.690, 95% CI [1.195–2.391]), while 32–34 weeks showed a 47.7% elevated risk (adjusted OR = 1.477, 95% CI [1.045–2.088]). After removing the variable “history of PTB” from the set of adjusted confounding factors, the results remained virtually unchanged, suggesting the findings were robust. Similar results were also obtained in the subgroups based on gestational weeks. Notably, during the third trimester, per 2 g decrease in daily PUFAs intake was associated with a progressive increase in PTB risk, with the trend test reaching statistical significance (*P* = 0.047).

**Figure 3:**
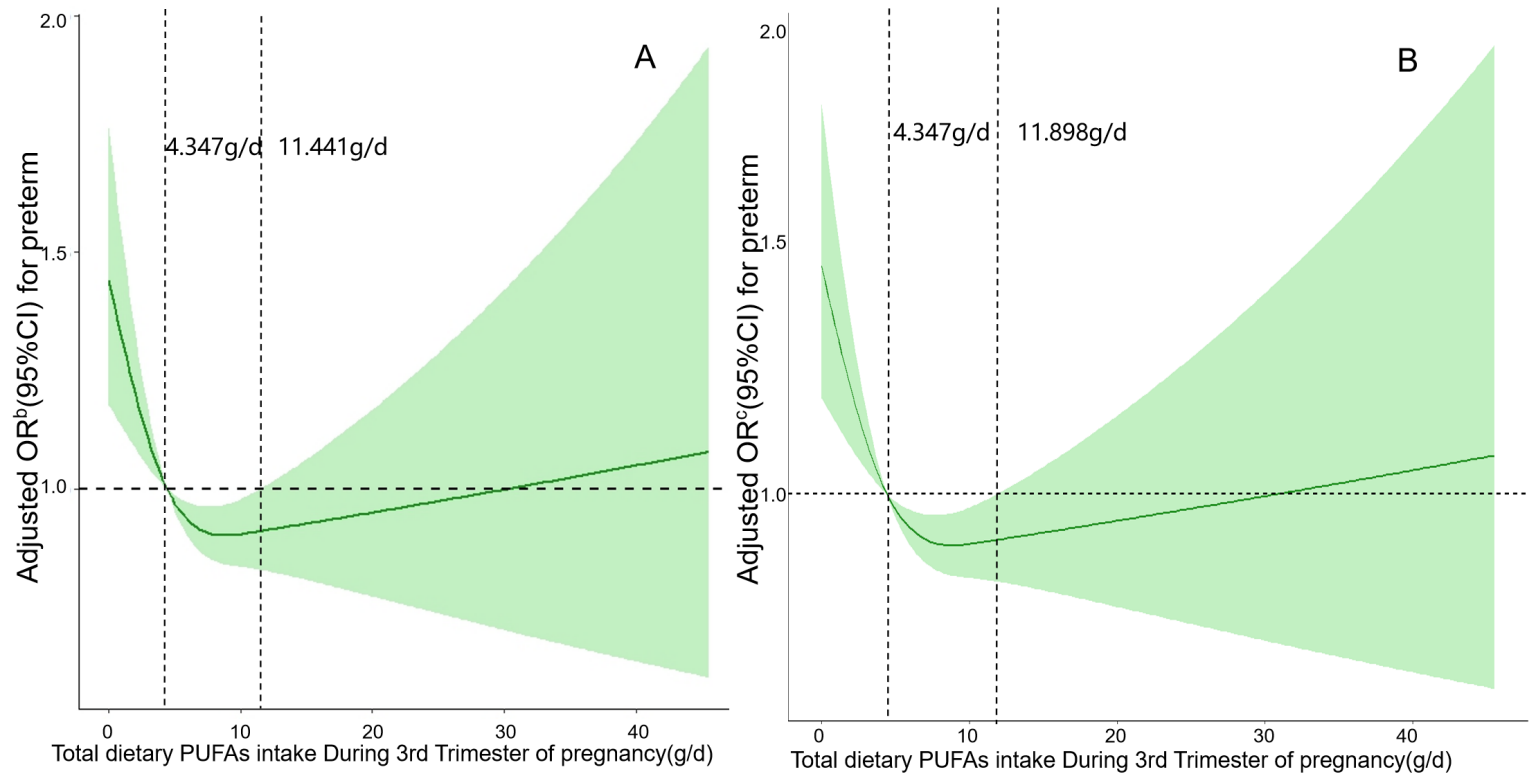
(A) and (B) respectively depict restricted cubic spline curves of the association between PUFAs intake and the risk of PTB at the third trimester before and after excluding a history of preterm birth.

Figures 3A and 3B respectively depict restricted cubic spline curves of the association between PUFAs intake and the risk of PTB at the third trimester before and after excluding a history of PTB. We observed a U-shaped association between the intake of PUFAs in the diet and the risk of PTB (*P*_Nonlinear_ < 0.05), meaning that both too low and too high intake of PUFAs in the diet will increase the risk of PTB. In the third trimester of pregnancy, when the daily intake is within the range of (4.347–11.441) g and (4.347–11.898) g, PUFAs may have a protective effect on PTB. When the daily intake of PUFAs is less than 4.347 g or higher than 11.441 g and 11.898 g, the risk of PTB has significantly increased.

### Interaction effects of maternal dietary vitamin A and PUFAs intake on the risk of preterm

As detailed in Table 4, we investigated the multiplicative interactions between maternal dietary vitamin A and PUFAs intake levels on PTB risk. Significant multiplicative interactions were observed both in the preconception period (*OR* = 1.232, 95% CI [1.032–1.471]) and across the entire gestation (*OR* = 1.396, 95% CI [1.158–1.683]), with statistically significant trend test results (*P* = 0.0208 and *P* < 0.0005, respectively). Stratified analyses by pregnancy trimesters yielded consistent findings. Notably, during the third trimester, the combined effects of vitamin A and PUFAs intake on PTB risk became more pronounced: (1) Low vitamin A and low PUFAs: Associated with a 27.2% increased risk of PTB (95% CI [1.176–1.377]); (2) High vitamin A and Low PUFAs: Corresponding to a 26.6% elevated risk (95% CI [1.004–1.598]); (3) Low vitamin A and High PUFAs: Linked to an 18.9% higher risk (95% CI [1.074–1.317]). After excluding history of PTB from the analysis, the results remained virtually unchanged.

## Discussion

This nested case-control study comprehensively and systematically investigated the effects of dietary vitamin A and PUFAs intake levels preconception and during pregnancy on the risk of PTB, and revealed the dose–response relationship and interaction between these two nutrients on PTB. This study found that the intake of PUFAs in the third trimester was associated with the risk of PTB in a U-shaped pattern. Additionally, when the intakes of vitamin A and PUFAs were both low preconception and during pregnancy, there might be a multiplicative interaction with the risk of PTB. At present, there is limited research on the relationship between these two nutrients and their interaction and PTB. Therefore, it is necessary to conduct further studies to verify and clarify the above results.

**Table 3:** Associations of maternal dietary PUFAs intake with the risk of preterm.

Dietary PUFAs intake (g/d)		Term (8,017)	Preterm (880)	OR^a^ (95% CI)	OR^b^ (95% CI)	OR^c^ (95% CI)	32<gestweek (162)			32≤gestweek<34 (170)			34≤gestweek<37 (548)		
							Cases	OR^b^(95% CI)	OR^c^(95% CI)	Cases	OR^b^(95% CI)	OR^c^(95% CI)	Cases	OR^b^(95% CI)	OR^c^(95% CI)
Preconception	<1.43	2,040	298	1.500 (1.293–1.740)	1.143 (0.973–1.343)	1.149 (0.980–1.348)	53	0.995 (0.701–1.413)	0.989 (0.698–1.401)	67	1.360 (0.977–1.891)	1.362 (0.980–1.893)	178	1.143 (0.936–1.394)	1.150 (0.944–1.402)
	≥1.43	5,977	582	1.000	1.000	1.000	109	1.000	1.000	103	1.000	1.000	370	1.000	1.000
	Per 2g decrease			1.149 (1.081–1.221)	1.029 (0.961–1.101)	1.028 (0.961–1.100)		0.951 (0.818–1.107)	0.952 (0.819–1.106)		1.002 (0.861–1.165)	1.008 (0.867–1.171)		1.060 (0.976–1.151)	1.062 (0.978–1.152)
	P for trend			<.0001	0.414	0.414		0.517	0.520		0.980	0.920		0.169	0.154
First trimester	<4.20	3,627	488	1.507 (1.310–1.733)	1.112 (0.948–1.304)	1.117 (0.954–1.309)	95	1.121 (0.792–1.587)	1.101 (0.779–1.557)	100	1.139 (0.810–1.600)	1.143 (0.813–1.605)	293	1.107 (0.910–1.346)	1.112 (0.915–1.352)
	≥4.20	4,390	392	1.000	1.000	1.000	67	1.000	1.000	70	1.000	1.000	255	1.000	1.000
	Per 2g decrease			1.175 (1.115–1.239)	1.017 (0.957–1.082)	1.025 (0.964–1.089)		0.976 (0.851–1.119)	0.981 (0.857–1.124)		1.039 (0.908–1.190)	1.049 (0.916–1.200)		1.023 (0.949–1.103)	1.030 (0.956–1.110)
	P for trend			<.0001	0.586	0.434		0.722	0.786		0.578	0.489		0.556	0.436
Second trimester	<3.93	3,346	457	1.508 (1.312–1.734)	1.094 (0.932–1.283)	1.095 (0.934–1.283)	86	0.996 (0.706–1.407)	0.974 (0.691–1.373)	99	1.315 (0.935–1.848)	1.314 (0.935–1.847)	272	1.063 (0.872–1.295)	1.062 (0.872–1.293)
	≥3.93	4,671	423	1.000	1.000	1.000	76	1.000	1.000	71	1.000	1.000	276	1.000	1.000
	Per 2g decrease			1.178 (1.118–1.241)	1.023 (0.962–1.089)	1.028 (0.967–1.093)		0.952 (0.831–1.090)	0.951 (0.831–1.089)		1.069 (0.933–1.223)	1.076 (0.940–1.231)		1.031 (0.956–1.112)	1.035 (0.960–1.116)
	P for trend			<.0001	0.465	0.381		0.476	0.471		0.337	0.287		0.431	0.369
Third trimester	<2.03	1,561	277	1.900 (1.631–2.213)	1.319 (1.113–1.563)	1.340 (1.133–1.586)	64	1.690 (1.195–2.391)	1.706 (1.209–2.409)	60	1.477 (1.045–2.088)	1.509 (1.070–2.129)	153	1.155 (0.932–1.430)	1.178 (0.952–1.456)
	≥2.03	6,456	603	1.000	1.000	1.000	98	1.000	1.000	110	1.000	1.000	395	1.000	1.000
	Per 2g decrease			1.216 (1.153–1.281)	1.064 (1.001–1.132)	1.069 (1.006–1.136)		1.137 (0.987–1.309)	1.137 (0.988–1.309)		1.099 (0.961–1.258)	1.107 (0.968–1.266)		1.036 (0.962–1.117)	1.041 (0.967–1.121)
	P for trend			<.0001	0.047	0.032		0.076	0.072		0.169	0.137		0.346	0.290

**Notes.**

OR^a^ univariate analyses.

OR^b^ adjusted for weight gain during pregnancy, mother’s BMI, mother’s nation, mother’s age, gestational diabetes, total energy intake, monthly income per capita, maternal education level, smoking, maternal employ, multivitamin supplement, gestational hypertension, history of premature birth, reproductive history and dietary vitamin A intake.

OR^c^ was adjusted for the factors included in OR^b^, with the history of premature birth excluded.

**Table 4:** Interaction effects of maternal dietary vitamin A and PUFAs intake on the risk of preterm.

Maternal dietary intake	Term/Preterm	OR^a^ (95% CI)	OR^b^ (95% CI)	OR^c^ (95% CI)
Preconception				
High VA and high PUFAs	3,129/264	1.000	1.000	1.000
High VA and low PUFAs	654/78	1.414 (1.083–1.845)	1.161 (0.873–1.544)	1.161 (0.875–1.539)
Low VA and high PUFAs	2,848/318	1.150 (1.056–1.253)	1.106 (1.007–1.215)	1.086 (1.084–1.087)
Low VA and low PUFAs	1,386/220	1.234 (1.159–1.315)	1.087 (1.010–1.170)	1.085 (1.009–1.166)
Multiplicative interaction: OR^b^ (95% CI) = 1.232 (1.032–1.471), *P* < 0.0208 OR^b^ (95% CI) = 1.235 (1.036–1.472), *P* < 0.0186				
During pregnancy				
High VA and high PUFAs	4,321/357	1.000	1.000	1.000
High VA and low PUFAs	1,594/186	1.413 (1.173–1.702)	1.095 (0.891–1.346)	1.106 (0.900–1.358)
Low VA and high PUFAs	953/131	1.290 (1.160–1.434)	1.181 (1.049–1.329)	1.177 (1.046–1.325)
Low VA and low PUFAs	1,149/206	1.295 (1.218–1.376)	1.164 (1.079–1.256)	1.158 (1.074–1.249)
Multiplicative interaction: OR^b^ (95% CI) = 1.396 (1.158–1.683), *P* < 0.0005 OR^b^ (95% CI) = 1.369 (1.137–1.649), *P* < 0.0009				
First trimester				
High VA and high PUFAs	3,650/294	1.000	1.000	1.000
High VA and low PUFAs	2,167/247	1.415 (1.185–1.690)	1.155 (0.950–1.405)	1.168 (0.962–1.419)
Low VA and high PUFAs	740/98	1.282 (1.136–1.447)	1.173 (1.026–1.342)	1.169 (1.022–1.336)
Low VA and low PUFAs	1,460/241	1.270 (1.196–1.349)	1.119 (1.034–1.211)	1.128 (1.127–1.129)
Multiplicative interaction: OR^d^ (95% CI) = 1.313 (1.100–1.566), *P* < 0.0025 OR^b^(95% CI) = 1.318 (1.105–1.572), *P* < 0.0021				
Second trimester				
High VA and high PUFAs	3,888/313	1.000	1.000	1.000
High VA and low PUFAs	1,973/228	1.436 (1.200–1.717)	1.136 (0.931–1.385)	1.139 (0.935–1.388)
Low VA and high PUFAs	783/110	1.321 (1.177–1.482)	1.191 (1.048–1.355)	1.183 (1.041–1.345)
Low VA and low PUFAs	1,373/229	1.275 (1.200–1.354)	1.159 (1.073–1.252)	1.155 (1.070–1.247)
Multiplicative interaction: OR^b^ (95% CI) = 1.342 (1.124–1.602), *P* < 0.0012 OR^b^ (95% CI) = 1.314 (1.098–1.572), *P* < 0.0028				
Third trimester				
High VA and high PUFAs	5,068/419	1.000	1.000	1.000
High VA and low PUFAs	866/122	1.704 (1.376–2.111)	1.266 (1.004–1.598)	1.288 (1.023–1.622)
Low VA and high PUFAs	1,388/184	1.266 (1.155–1.388)	1.189 (1.074–1.317)	1.176 (1.063–1.301)
Low VA and low PUFAs	695/155	1.392 (1.302–1.488)	1.272 (1.176–1.377)	1.270 (1.174–1.373)
Multiplicative interaction: OR^b^ (95% CI) =1.680 (1.362-2.071), *P* < 0.0001 OR^b^(95% CI) = 1.672 (1.359–2.059), *P* < 0.0001				

**Notes.**

OR^a^ univariate analyses.

OR^b^ adjusted for weight gain during pregnancy, mother’s BMI, mother’s nation, mother’s age, gestational diabetes, total energy intake, monthly income per capita, maternal education level, smoking, maternal employ, multivitamin supplement, gestational hypertension, history of premature birth, reproductive history.

OR^c^ was adjusted for the factors included in OR^b^, with the history of premature birth excluded.

A meta-analysis showed that vitamin A supplements during pregnancy effectively reduced the risk of abnormal growth and birth outcomes, including the risk of PTB (overall reduced by 19%), the risk of LBW (overall reduced by 16%) and the risk of anemia (overall reduced by 20%), and increased the weight and height of newborns (Ma et al., 2023). A phased randomized clinical trial based on the Bayesian model indicates that supplementing DHA during pregnancy can reduce the risks of PTB and newborns’ admission to the NICU, and the total intake of vitamin A may be related to prolonging the pregnancy period (Wang et al., 2023). A comprehensive review of relevant literature indicated that supplementing with EPA and DHA (with particularly strong evidence for DHA alone) had a positive effect in reducing the risk of PTB, especially early preterm birth (EPTB). Two ongoing large-scale studies should further clarify and confirm whether DHA can be used in clinical practice, and recommended for all pregnant women or specific risk groups (Samuel et al., 2019; Baker et al., 2024). A randomized controlled trial showed that low levels of total ω-3 PUFAs in early pregnancy were associated with a high risk of PTB. Among women with a total ω-3 status ≤4.1% of total fatty acids, ω-3 supplements substantially reduced the risk of early PTB compared with control (0.73% vs.3.16%; relative risk = 0.23, 95% CI [0.07–0.79]) (Cetin et al., 2024; Simmonds et al., 2020). A cohort study showed that in the Australian indigenous women’s cohort, the concentrations of EPA and DHA in the red blood cell membranes of women with premature birth were relatively low. Moreover, a higher dietary intake of long-chain ω-3 PUFAs was associated with a lower risk of PTB and preeclampsia (Gray et al., 2023).

Maternal nutrition intake during pregnancy is vital for the health of both the mother and the baby, directly influencing fetal development, the mother’s physiological adaptation, and long-term health. Vitamin A (retinol), as a crucial fat-soluble antioxidant, exhibits a strong correlation between its molecular structural characteristics and antioxidant function. The molecule consists of a β-ionone ring and an isoprenoid side chain containing four double bonds, with its distinctive conjugated diene structure (C5-C6 and C7-C8 conjugated double bonds) conferring potent antioxidant properties (Carazo et al., 2021). It plays an essential role in maintaining the function of the placenta, promoting fetal development, and reducing intrauterine infections. PUFAs are characterized by the presence of two or more carbon–carbon double bonds along their carbon chains. PUFAs are categorized into two main types: ω-3 and ω-6 fatty acids. These fatty acids exhibit a broad spectrum of biological activities and play vital physiological roles, such as contributions to the structure of human cell membranes and modulation of inflammatory and immune responses (Kapoor et al., 2021). Its active conjugated double bond can consume reactive oxygen species (ROS) through preferential self-oxidation, thereby interrupting the lipid peroxidation chain reaction (Lewicka et al., 2017); at the same time, it can directly eliminate various highly reactive free radicals such as singlet oxygen (^1^O_2_), hydroxyl radicals (⋅OH), *etc.* (Kołodziejczyk & Karwowski, 2025); moreover, due to its lipophilic property, this compound can embed in the phospholipid bilayer of the cell membrane, providing direct and clearly localized protection for membrane structures rich in PUFAs (Redfern, 2020; Lewicka et al., 2017; Bi et al., 2023; Ciaccio et al., 1993).

Under conditions of vitamin A deficiency during pregnancy, the impairment of these protective mechanisms triggers multiple pathological alterations (Petiz et al., 2017). Firstly, diminished radical scavenging capacity enables ROS to attack methylene-interrupted double bonds in ω − 3/ω − 6 PUFAs within cell membranes, initiating lipid peroxidation cascades and leading to toxic end-product accumulation such as malondialdehyde (MDA) (Rochette et al., 2022; Oladele et al., 2025). Secondly, aberrant activation of the nuclear factor κB (NF-κB) signaling pathway promotes excessive production of pro-inflammatory cytokines (TNF-α, IL-6, *etc.*), disrupting immune homeostasis (Pandey et al., 2000; Wang et al., 2015; He & Karin, 2011; Raverdeau & Mills, 2014; Dolin et al., 2023; Bono et al., 2016). Notably, vitamin A and PUFAs (particularly ω-3 series) synergistically regulate the NF-κB phosphorylation cascade to suppress inflammatory mediator release. This coordinated molecular interaction may be the underlying mechanism for the observed interaction between vitamin A and PUFAs in current research. In summary, vitamin A and PUFAs may play important roles in modulating the risk of PTB, potentially through mechanisms involving anti-inflammatory effects, immune regulation, and placental function maintenance.

Based on the Dietary Reference Intakes for China (2023 Edition), our research found that 25.04% of pregnant women reached the recommended intake of vitamin A in the third trimester. This study indicated that low intake of vitamin A preconception and during pregnancy may increase the risk of PTB. This might be due to the antioxidant and anti-inflammatory effects of vitamin A and its derivatives. When the level of vitamin A in a pregnant woman’s body is insufficient, her body’s antioxidant and anti-inflammatory capabilities may decline, thus potentially increasing the risk of PTB. Previous studies have also shown that excessive intake of vitamin A during pregnancy may increase the risk of fetal abnormalities (Abadie et al., 2023; Olson, Ameer & Goyal, 2023). These findings highlight the need for a balanced intake of vitamin A during pregnancy. Our research has not yet identified a significant association between excessive vitamin A intake and PTB. Therefore, a larger sample size and more precise exposure assessment may be necessary to validate these findings. Furthermore, the “Dietary Reference Intakes for China (2023 Edition)” provides the reference intake ranges for ω-3 and ω-6 PUFAs. This study found that PUFAs may have a U-shaped association with the risk of PTB. Both low and high intake levels of PUFAs may increase the risk of PTB. Low levels of PUFAs may lead to fetal growth restriction, placental dysfunction or dysregulation of inflammatory responses, and might increase the risk of PTB. On the contrary, high levels of PUFAs may trigger oxidative stress, dysregulation of prostaglandin metabolism (such as elevated prostaglandin E2 levels) or excessive immune activation, and might also increase the risk of PTB. In contrast, moderate PUFAs levels are associated with the lowest risk of PTB. In conclusion, implementing optimized nutritional intervention strategies during pregnancy may help reduce the occurrence of PTB. For instance, appropriate supplementation of vitamin A and adjustment of the intake ratio of PUFAs can be considered. This study was a large-sample nested case-control study, with the main research subjects being women in the urban area of Lanzhou. The dietary nutrient intake levels of these subjects were not substantially different from those of women in other western Chinese cities. Therefore, the study subjects remained representative of the female population in western Chinese cities and can provide a reference for related research. Future research should further explore the feasibility of personalized nutritional intervention and clarify its effectiveness in different populations.

## Strengths and Limitations

This study had several strengths and limitations that should be acknowledged. This study was based on a large-scale prospective birth cohort with complete follow-up, providing high-quality evidence for the conclusion. By prospectively collecting dietary data of pregnant women during different pregnancy periods, the dynamic association between vitamin A and PUFAs intake and PTB was systematically analyzed. Moreover, for the first time, the interaction between these two nutrients and PTB at different pregnancy stages was explored, providing a new perspective for understanding the complex influence of nutritional factors. However, this study also had certain limitations. First, all data were obtained through participant questionnaires, which may introduce recall bias. To minimize for recall bias, in both the field investigation and data entry processes of this study, systematic and strict quality control measures were implemented. Second, quantitative analysis of interactions between multiple dietary nutrients was not feasible, potentially leading to residual confounding. We collected detailed information such as demographic characteristics, medical history and lifestyle to adjust for potential confounding factors. Through strict quality control, multivariate logistic regression and stratified analysis, we attempted to control the confounding effects as much as possible. Additionally, although there was a possibility of misclassification bias, we verified the diagnosis of PTB through medical records to minimize such bias to the greatest extent.

## Conclusions

This study indicates that insufficient intake of vitamin A and PUFAs preconception and during pregnancy may increase the risk of PTB. Furthermore, both excessive and insufficient intake of PUFAs during the third trimester may increase the risk of PTB. Therefore, it is recommended that pregnant women pay attention to consuming foods rich in vitamin A and PUFAs preconception and during pregnancy, such as deep-sea fish, animal-based foods, and dairy products. This may help reduce the risk of PTB.

##  Supplemental Information

10.7717/peerj.21068/supp-1 Supplemental Information 1 Raw Data

10.7717/peerj.21068/supp-2 Supplemental Information 2 STROBE checklist

## Additional Information and Declarations

### Data Availability

The following information was supplied regarding data availability: The raw measurements are available in the Supplementary File.
